# DNA methylation is maintained with high fidelity in the honey bee germline and exhibits global non-functional fluctuations during somatic development

**DOI:** 10.1186/s13072-019-0307-4

**Published:** 2019-10-10

**Authors:** Keith D. Harris, James P. B. Lloyd, Katherine Domb, Daniel Zilberman, Assaf Zemach

**Affiliations:** 1 School of Plant Sciences and Food Security, Tel-Aviv University, 69978 Tel-Aviv, Israel; 2 Center for RNA Systems Biology, University of California, Berkeley, Berkeley, CA 94720 USA; 3 Present Address: ARC Centre of Excellence in Plant Energy Biology, The University of Western Australia, Perth, WA 6009 Australia; 4 Department of Cell and Developmental Biology, John Innes Center, Norwich, UK

**Keywords:** Epigenetics, DNA methylation, Gene body methylation, Non-CG methylation, DNMT3, Germline

## Abstract

**Background:**

DNA methylation of active genes, also known as gene body methylation, is found in many animal and plant genomes. Despite this, the transcriptional and developmental role of such methylation remains poorly understood. Here, we explore the dynamic range of DNA methylation in honey bee, a model organism for gene body methylation.

**Results:**

Our data show that CG methylation in gene bodies globally fluctuates during honey bee development. However, these changes cause no gene expression alterations. Intriguingly, despite the global alterations, tissue-specific CG methylation patterns of complete genes or exons are rare, implying robust maintenance of genic methylation during development. Additionally, we show that CG methylation maintenance fluctuates in somatic cells, while reaching maximum fidelity in sperm cells. Finally, unlike universally present CG methylation, we discovered non-CG methylation specifically in bee heads that resembles such methylation in mammalian brain tissue.

**Conclusions:**

Based on these results, we propose that gene body CG methylation can oscillate during development if it is kept to a level adequate to preserve function. Additionally, our data suggest that heightened non-CG methylation is a conserved regulator of animal nervous systems.

## Background

Cytosine methylation is an ancient DNA modification that regulates the functioning of eukaryotic genomes [1, 2]. Methylation can be epigenetically inherited, most classically through the action of the Dnmt1 methyltransferase that maintains methylation within symmetrical CG dinucleotides in plant and animal genomes [3]. Inheritance of DNA methylation patterns across generations is robust and well-established in plants, but variable and controversial in mammals [4–6]. Plants and animals also have Dnmt3-family methyltransferases that establish new methylation patterns and contribute to the maintenance of existing ones [1]. Methylation in animal genomes has long been thought to exist exclusively within the CG context, but recent work has revealed the presence of Dnmt3-catalyzed non-CG methylation in specific mammalian cell types and tissues, particularly in the brain [7, 8].

DNA methylation represses transposable elements in plants, vertebrates, fungi, and likely other species [9–11]. Methylation of promoters and other gene regulatory elements also generally causes transcriptional silencing, and this type of methylation is modulated to regulate gene expression and development in plants and vertebrates [2, 12]. More enigmatic targets of methylation are the transcribed portions of genes, a phenomenon known as gene body methylation [13]. In animals and flowering plants, gene body methylation is enriched in the exons of highly conserved genes that are ubiquitously and moderately transcribed, e.g. housekeeping genes that are constitutively expressed in all cell types and under diverse conditions [14–28]. These associations suggest a similar biological function, and possibly a common evolutionary origin, for gene body methylation in animals and plants. Extensive gene body methylation in the green algae *Chlorella variabilis* and *Klebsormidium nitens* [11, 29] also suggests a common evolutionary origin. Other findings, including the lack of gene body methylation in early-branching land plants and the linkage of genic methylation with a plant-specific DNA methyltranferase, support a convergent evolutionary model, in which gene body methylation evolved separately in animals and plants [30–33].

As a robust epigenetic feature that targets thousands of coding sequences in many eukaryotic genomes [9, 10], gene body methylation is expected to have an important function [34], especially because cytosine methylation is known to be mutagenic and should therefore be disfavored in coding sequences [35, 36]. Since its discovery, extensive efforts have been made to investigate the molecular and developmental roles of gene body methylation. Gene body methylation was found to fluctuate during animal and plant development [37–40], and several studies have linked artificial or developmental changes in gene body methylation to altered expression of particular genes [41–43]. Gene body methylation was also reported to regulate the splicing of individual genes [43–46]. However, DNA methyltransferase mutants in which methylation within gene bodies is nearly eliminated show no obvious global expression changes of body-methylated genes [28, 31, 32, 47], suggesting that gene body methylation in general either functions downstream of transcription [32, 48] or that its effects on transcription are subtle and difficult to detect by standard RNA sequencing techniques [34]. For example, the association between gene body methylation and constitutive gene expression could imply a role in homeostatic regulation of transcription, such as transcriptional stabilization or repression of unstable aberrant transcripts [49, 50]. Indeed, Dnmt3-mediated methylation of actively-transcribed genes was recently shown to inhibit aberrant intragenic transcriptional initiation in mouse cells [51], and gene body methylation has been linked with suppression of aberrant antisense transcripts in *Arabidopsis* [52].

Developmental or experimental changes in gene body methylation within vertebrate and plant genomes occur in the context of altered methylation in transposons and gene regulatory sequences [53, 54], which complicates assignment of functional outcomes specifically to changes in gene body methylation. For this reason, honey bees and other invertebrates, in which methylation is specifically targeted to gene bodies, are highly useful models that provide a direct link between genic methylation and phenotype [9, 14]. Knockdown of Dnmt3 in honey bees shifted the development of worker larvae into adult queens [55], implying that an artificial reduction of gene body methylation can alter development. However, it is still unclear whether gene body methylation actively participates in executing developmental pathways in bees and other insects. Dynamic changes in the level and distribution of methylation during development would be consistent with an active role in developmental regulation. Several studies have reported that gene body methylation patterns in honey bee and other insects differ between developmental stages [43, 56, 57]. However, the robustness and biological reproducibility of the reported changes have been recently questioned [58].

**Fig. 1:**
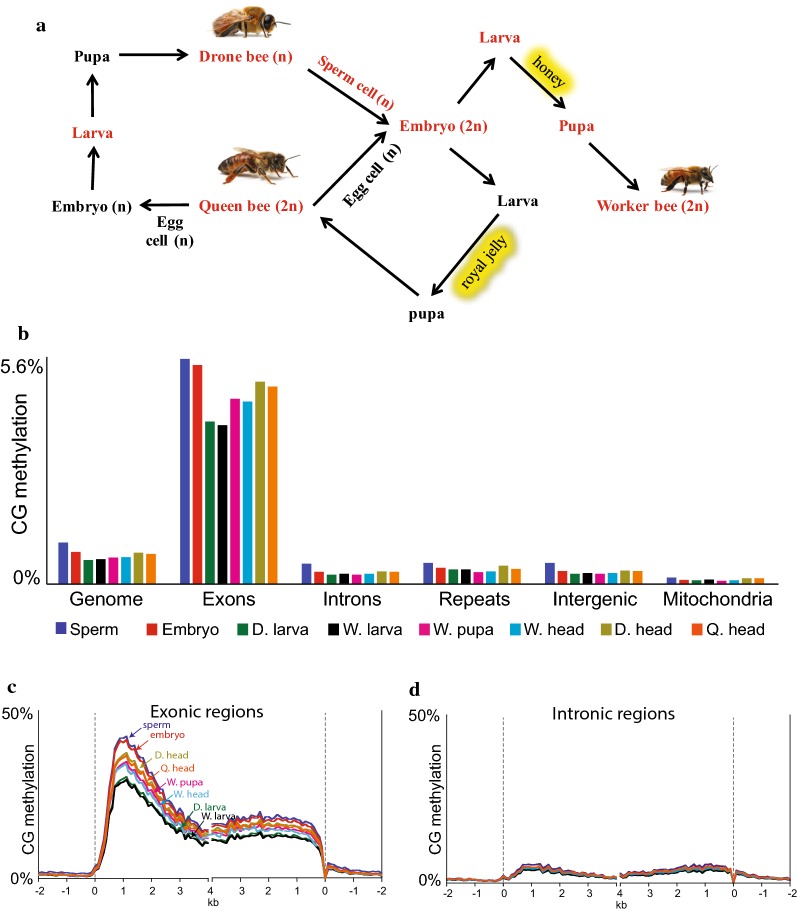
Gene body methylation greatly fluctuates during honey bee development. **a** Schematic illustration of honey bee life cycle. Developmental stages examined in this study are in red. **b** Averaged CG methylation levels in the whole genome and specific annotations. Intergenic represents genomic sequences annotated as neither exonic, intronic, nor repeats. **c**, **d** Patterns of CG methylation in gene bodies in different developmental stages. Honey bee genes were aligned either at the 5′ or 3′ ends, and methylation of CG cytosines located specifically either in exons (**c**) or introns (**d**) were averaged within 100-bp intervals. The dashed lines represent points of alignment

To investigate DNA methylation dynamics during insect development, we profiled and analyzed the methylomes and transcriptomes derived from eight honey bee developmental stages (Fig. 1a). Using these biologically replicated data sets, we found that although the average methylation level of most genes alters substantially during honey bee development, very few sequences are specifically methylated in a particular developmental stage. Furthermore, DNA methylation changes are highly correlated between developmental stages. Fidelity of CG methylation is highest in sperm cells and embryos, the latter also exhibiting highest expression of Dnmt1. We did not find a significant association between methylation changes and developmentally regulated gene expression or alternative splicing. These results indicate that although the global levels of gene body methylation fluctuate during honey bee development, the overall methylation patterns are robustly maintained, suggesting that the main function of gene body methylation is homeostatic. The high methylation fidelity in sperm and the reported stability of methylation in wasp hybrids [59] suggest that gene body methylation in bees and related insects is trans-generationally inherited through robust germline maintenance, whereas methylation efficiency is lower and more variable during somatic development. Finally, we identified Dnmt3-associated non-CG methylation in the gene bodies of adult honey bee heads, suggesting that this may be an ancient and conserved feature of animal neurological development [7].

## Results

### Gene body methylation fluctuates during honey bee development

To investigate the dynamics of DNA methylation during honey bee development, we profiled the genome-wide methylomes of eight developmental stages: sperm, worker embryo, worker larva, drone larva, worker pupa, worker head, drone head, and queen head (Fig. 1a). We conducted two independent experiments (experiments 1 and 2, Additional file 1: Figure S1A), collecting material from each of the developmental stages about 3 weeks apart. Quantification of the averaged CG methylation level of the two biological replicates, either separately (Additional file 1: Figure S1A) or combined (Fig. 1b), in the whole nuclear genome, as well as within specific genetic elements, including exons, introns, and repeats, revealed two main characteristics of DNA methylation in honey bee. First, methylation is targeted specifically to exonic sequences in all developmental stages (Fig. 1b–d and Additional file 1: Figure S1). Average CG methylation for the whole genome is around 1%, within all exons is about 5%, and within the set of methylated exons (~ 25% of all exons) reaches approximately 40% (Fig. 1b and Additional file 1: Figure S1A). Second, the genomic average of gene body methylation within exons fluctuates during honey bee development. Exon methylation is high in the sperm and embryo, drops by about 25% in drone and worker larvae, and then increases to an intermediate level in pupa and adult heads (Fig. 1b and Additional file 1: Figure S1A). Meta-analyses of averaged exonic or intronic methylation across honey bee genes confirmed the above global methylation dynamics, further showing that exonic methylation changes across the entire gene sequence (Fig. 1c, d and Additional file 1: Figure S1B).

### Developmental methylation differences are consistent between diverse biological replicates

**Fig. 2:**
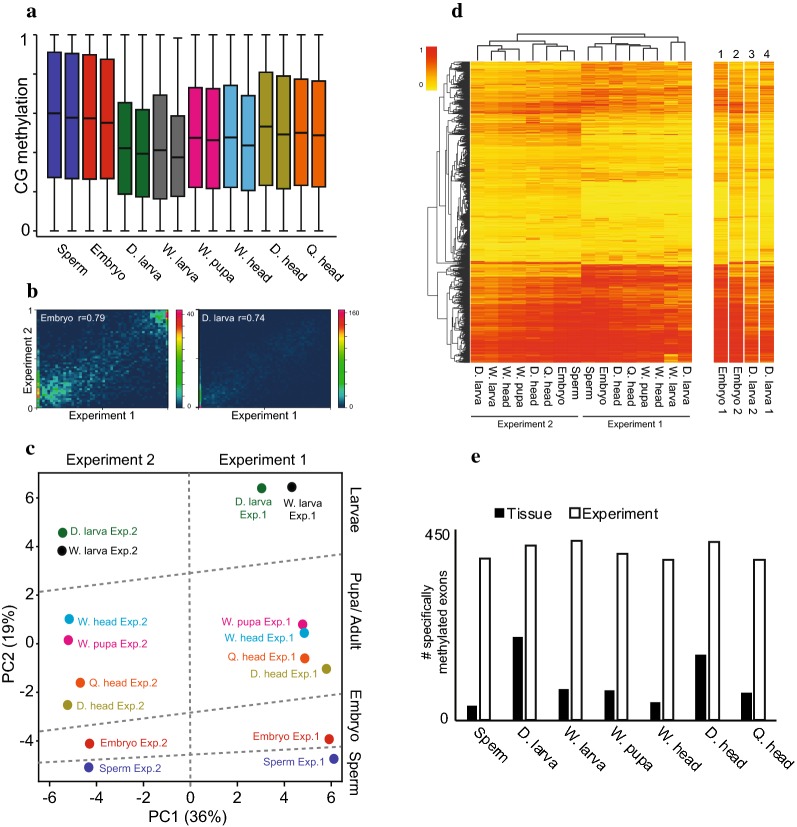
Experimental versus tissue methylation differences. **a** Box plots of CG methylation distribution within methylated exons in each of the developmental stages separated for experiment 1 (left box of each sample) and experiment 2 (right boxes). Methylated exons have a minimum 10% average methylation in either of the experiments. **b** Density scatter plots of methylation level in methylated exons (defined in **a**) correlated between biological replicates. *r* is Pearson correlation coefficient value (*p* < 10^−4^). **c** PCA based on methylation level in methylated exons from all replicates. Samples are clustered to experiments (PC1) and tissues (PC2). **d** Hierarchical clustering of methylation in methylated exons of all replicates. Heatmap scale of fractional methylation is stretched from 0 (yellow) to 1 (red). **e** Bar graphs show the number of exons methylated in embryo and the indicated sample only in one of the experiments (white bars) or one of the tissues (black bars). For example, the black bar in D. larva shows the number of exons methylated only in D. larva or in embryo (by comparing columns 1–2 to 3–4 in **d**), and D. larva’s white bar shows the number of exons methylated only in embryo and D. larva from experiment 1 or in embryo and D. larva from experiment 2 (by comparing columns 1 and 4 to columns 2–3 in **d**)

Meta-analyses of exon methylation showed consistent methylation levels between biological replicates (Fig. 2a). However, methylation of individual exons shows only moderate correlation between biological replicates, with Pearson’s correlation coefficients ranging between 0.65 and 0.79 (Fig. 2b and Additional file 1: Figure S2). Principal component analysis (PCA) separated methylation datasets by experiment in the first principal component (PC1), grouping datasets of experiment 1 away from those of experiment 2 (Fig. 2c). Similar separation by experiment was produced by hierarchical clustering (Fig. 2d). PCA analysis separated datasets by tissue along the second principal component (PC2), with tissues ordered roughly by the amount of overall methylation (compare Fig. 2a, c). Hierarchical clustering also grouped datasets by tissue methylation level, consistently separating samples with high methylation (sperm and embryo) from those with low methylation (larva; Fig. 2d). Comparison of methylation patterns in embryo and another tissue across experiments revealed many more exons specifically methylated in each experiment than in any tissue (Fig. 2e). These results demonstrate a greater divergence of methylation patterns between experiments than between tissues and illustrate the importance of biological replication for assessing tissue-specific methylation patterns in insects, as highlighted by an earlier study [58]. Biological replicates were collected at two separate time points (several weeks apart); thus, the experiment effect (Fig. 2c, d) could reflect a biological difference, e.g., seasonality. Comparison of methylation patterns between any tissue in experiment 1 and another in experiment 2 would identify many differentially methylated exons and create a false sense of extensive tissue-specific methylation. For the remainder of the study, a site is scored as differentially methylated between tissues only when comparisons within experiments 1 and 2 are in agreement and statistically significant.

### Genic CG methylation changes globally but not locally during honey bee development

Differential methylation between tissues could result from global and moderate fluctuations within many genic sequences, from strong fluctuations in a limited set of sequences, or from a combination of these mechanisms. To distinguish between these possibilities, we calculated the percentage change in methylation between the embryo and each of the other biological samples in three levels of resolution: individual cytosines, exons, and entire genes.

**Fig. 3:**
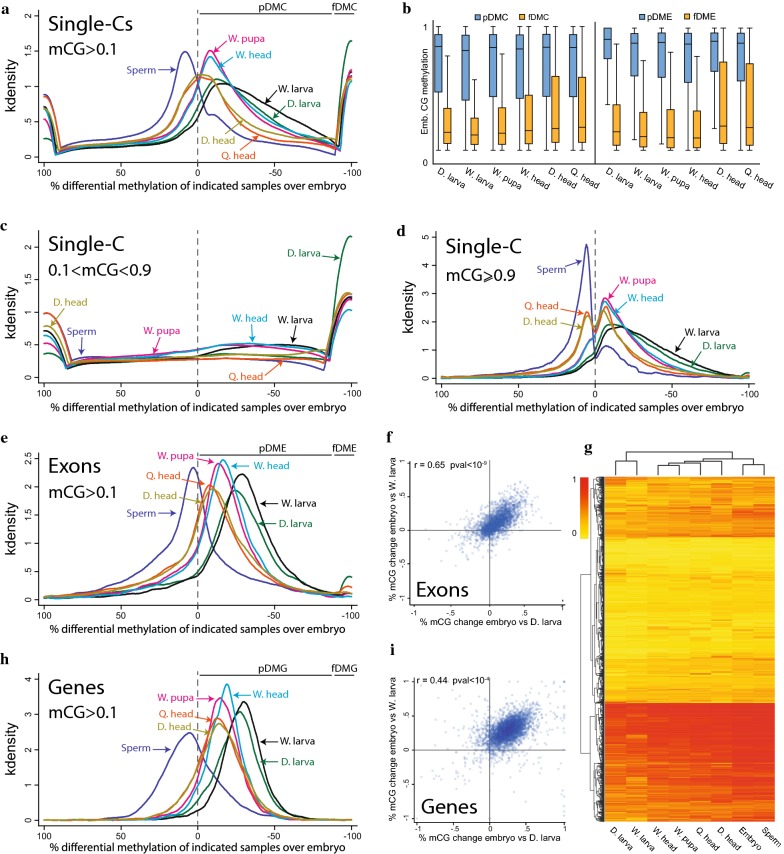
Gene body methylation is robustly maintained during honey bee development. **a** Kernel density plots of percent-methylation-change between averaged methylation in embryo versus indicated samples calculated for single cytosines (Single-Cs). Negative and positive numbers in the *x*-axis indicate greater and lower methylation in embryo and the other sample, respectively. Partial and fully differentially methylated cytosines (pDMC/fDMC) were considered if the percent-methylation-change was lower and higher than 90%, respectively. **b** Box plots of averaged embryonic methylation in single cytosines (left) and exons (right) derived from partially and fully differentially methylated sites between embryo and indicated samples. **c**, **d** Kernel density plots of percent-methylation-change between averaged methylation of single-Cs in embryo versus indicated samples, of cytosines derived from lowly methylated in embryo (**c**) or highly methylated in embryo (**d**). **e** Kernel density plots of percent-methylation-change between averaged methylation in embryo versus indicated samples calculated for single exons. Partial and fully differentially methylated exons (pDME/fDME) were annotated as in **a**. **f** Scatter plot of percent-methylation-change between embryo/D. larva and embryo/W. larva in exons. *r* is Pearson correlation coefficient value. **g** Hierarchical clustering heatmap showing average methylation in individual exons in the different biological samples. Scale bar indicates methylation level. **h** Kernel density plots of percent-methylation-change between averaged methylation in embryo versus indicated samples calculated for single genes. Partial and fully differentially methylated genes (pDMG/fDMG) were annotated as in **a**. **i** Scatter plot of percent-methylation-change between embryo/D. larva and embryo/W. larva in genes

Density plots of percent-methylation-change show that most individual methylated CG sites are hypermethylated in the sperm over the embryo, whereas in all postembryonic samples, most methylated CGs are hypomethylated compared to embryonic CGs (Fig. 3a). Furthermore, these plots show that differentially methylated cytosines (DMCs) can be divided into two groups: partial DMCs (< 90% change) and full DMCs (≥ 90% change; Fig. 3a). pDMCs outnumber fDMCs in all developmental pairwise comparisons (1.5- to 4.8-fold) (Additional file 1: Figure S3A). 22–44% of fDMCs between embryo and other tissues are shared between biological replicates (Additional file 1: Figure S3B), suggesting that at least some sites do lose all methylation during development. Nevertheless, we found that the level of CG methylation in embryos is much higher in pDMCs cytosines (median level of 95%) than in fDMCs cytosines (median level ~ 20%; Fig. 3b). Consistently, almost all fDMCs are methylated below 90% in the embryo, whereas pDMCs have methylation ≥ 90% (Fig. 3c, d). These results indicate that highly methylated embryonic cytosines are usually partially hypomethylated and very rarely lose all their methylation during development.

As gene body methylation in honey bee is localized specifically in exons (Fig. 1b–d), we next examined DNA methylation dynamics across individual exons during bee development. Most exons are partially hypomethylated in postembryonic samples, with a median hypomethylation ranging from 8% in adult heads to 29% in larval samples (Fig. 3e and Additional file 1: Figure S3C). Exon methylation changes between embryos and post-embryonic samples are correlated (Fig. 3f and Additional file 1: Figure S3C), so that a given exon tends to have an altered methylation level in the same direction and to a similar extent in all post-embryonic samples. This result is also illustrated in hierarchical clustering of methylation in exons, which segregated samples according to their overall exonic methylation level (Fig. 3g). On average, only 2.9% of the differentially methylated exons (181 exons) had completely lost their methylation in postembryonic samples, in contrast to an average of 6240 exons that were partially hypomethylated (Additional file 1: Figure S3A). Similar to individual cytosines, we found that fully differentially methylated exons are much less methylated in the embryo (median level of 21–27%) than exons with partial differential methylation (median level of 83–85%; Fig. 3b), suggesting that lowly methylated CG sites and exons are more easily fully demethylated than highly methylated sites and exons.

Finally, we found that very few genes (3–9 genes) completely lose their methylation during development (Additional file 1: Figure S3A), whereas most genes show methylation reduction between 13 and 30% (Fig. 3h and Additional file 1: Figure S3C). Similar to exons, genic methylation changes between embryos and post-embryonic samples are correlated (Fig. 3i and Additional file 1: Figure S3D), so that a given gene tends to change methylation in the same direction and to a similar extent in all post-embryonic samples.

In summary, our DNA methylation analyses show that global gene body methylation fluctuates during honey bee development, but specific differential methylation patterns at the level of complete genes or exons are rare. The patterns of gene body methylation in honey bee are, therefore, very robust and are largely maintained in distinct tissues throughout the bee life cycle.

### Gene body methylation dynamics are not generally associated with differential gene expression during bee development

**Fig. 4:**
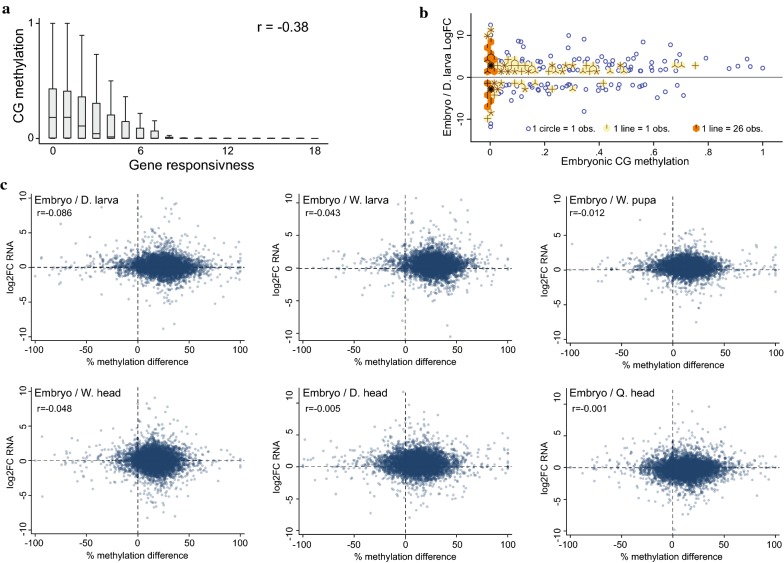
Gene body methylation dynamics are not associated with developmentally regulated transcriptional profiles. **a** Boxplot of averaged genic CG methylation in embryos over gene responsiveness (the breadth of expression of a gene across multiple developmental stages). Gene responsiveness was calculated using the complete transcriptome array of this study. A gene with responsiveness level of zero means that it was found to be similarly expressed in all developmental stages, whereas a gene with responsiveness level of 21 means that it was found to be differentially expressed in all pairwise comparisons among the seven developmental stages. **b** Sunflower (density) scatter plot of LogFC of RNA reads of DEGs between embryo and D. larva versus averaged genic CG methylation in embryos. Obs. equals observations. **c** Scatter plots of LogFC of RNA versus percent-methylation-change between indicated samples. *r* represents Pearson Correlation Coefficient value

Gene body methylation in honey bee, as well as in other animals and plants, was previously shown to be anticorrelated with gene responsiveness and to be enriched at ubiquitously expressed genes [14–17, 21–28]. We confirmed this finding using transcriptional profiles from all developmental stages examined in this study (Fig. 4a). Consistently, most differentially expressed genes (DEGs) between developmental stages are not methylated (Fig. 4b and Additional file 1: Figure S4A). In addition, unmethylated genes present a broader range of differential expression than methylated genes (Fig. 4b and Additional file 1: Figure S4A). The number of overlapping DEGs and differentially methylated genes (DMGs) is smaller (in most cases much smaller) than expected by random chance in all pairwise comparisons of the examined developmental stages (column 9 in Table 1). Therefore, both methylated and differentially methylated genes are underrepresented among developmentally regulated genes. Taken together with the above-described behavior of DNA methylation during bee development, these results suggest that gene body methylation does not function primarily to regulate developmentally-specialized transcriptional programs.

**Table 1:**
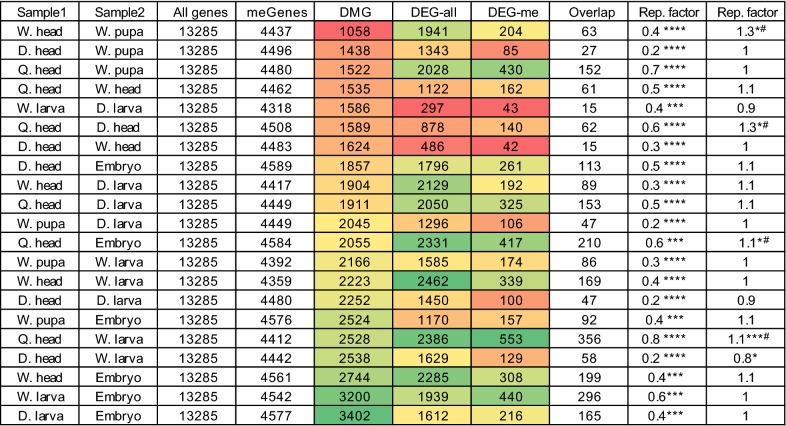
Gene body methylation dynamics are not associated with developmentally regulated transcriptional profiles

Table includes the names of the pairwise samples (columns 1–2), the number of all genes (column 3), number of methylated genes (> 0.1 in either of the selected samples; column 4), number of differentially methylated genes (averaged genic mCG > 0.1 in either sample, Fisher Exact Test pval < 0.05 in both of biological replicates; column 5), number of differentially expressed genes among either all genes or within methylated ones (columns 6 and 7, respectively), number of overlapped genes between DMG and DEG (column 8), and the ratio between the number of actual overlapped genes with that of the expected one when considering all genes (column 9) or only methylated ones (column 10)

* Denote the strength of *p* values. Brackets above the table point to columns used for calculating relevant representation factors

^#^Marks observations with a significant overlap between DMGs and DEGs among methylates genes

However, the global fluctuations in methylation during honey bee development might nonetheless influence developmentally-regulated transcription of methylated genes. To test this hypothesis, we focused our analyses specifically on differentially expressed methylated genes (DEG-me/column 7 in Table 1). We found that for the majority of the pairwise comparisons, including the ones with the highest methylation dynamics (i.e., embryo vs. larvae), the number of overlapping genes between DEGs and DMGs is just as expected by random chance if only methylated genes are considered (column 10 in Table 1). Thus, DEGs are not generally enriched among DMGs. Four pairwise comparisons did show a significantly enriched overlap between DMGs and DEGs, and all of these contained at least one adult bee head (Table 1, marked with #). To further test the relationship between DEGs and DMGs, we correlated the levels of methylation and RNA changes between all pairs of developmental stages. This analysis found no association between methylation and expression dynamics (Fig. 4c), including between the DEG/DMG enriched overlap samples (Additional file 1: Figure S4B). Overall, our results indicate that gene body methylation dynamics in honey bee are not generally associated with changes in gene expression. Neurological tissues may be an exception, with some evidence that differential methylation is linked to differential expression (Table 1).

### Gene body methylation dynamics are not associated with differential RNA splicing during bee development

**Fig. 5:**
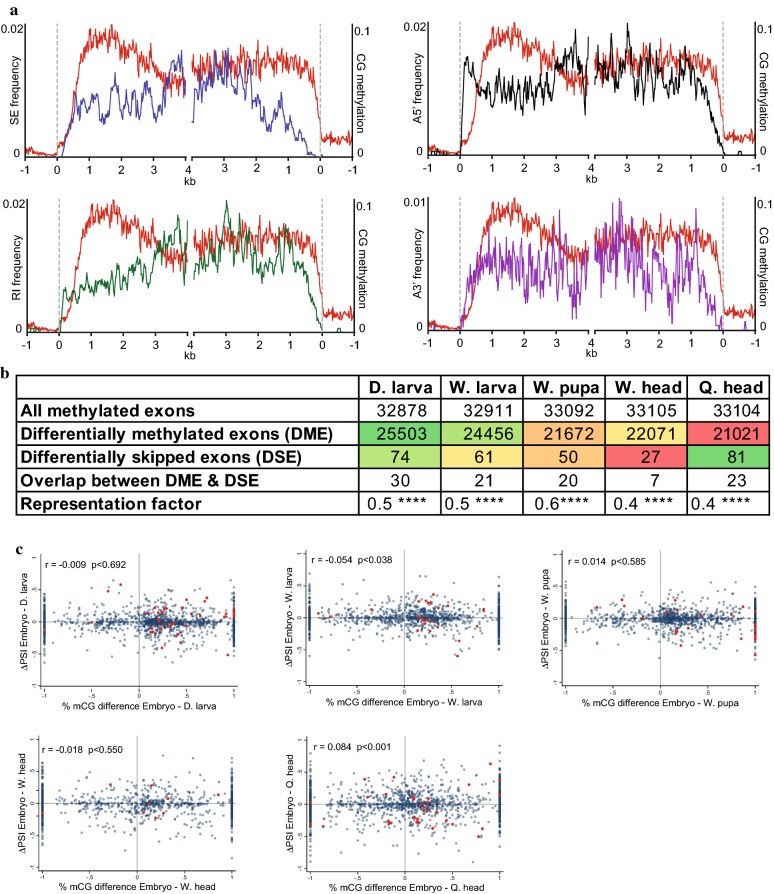
Gene body methylation dynamics are not associated with developmentally regulated RNA splicing patterns. **a** Skipped exons (blue), retained introns (green) and alternative 5′/3′ spliced sites (black/purple) frequencies were averaged in 20-bp bins along methylated genes (averaged genic methylation > 0.05) essentially as described in Fig. 1a. Averaged CG methylation profile is illustrated with red lines. **b** This table includes the number of all methylated exons (mCG > 0.05 in either embryo or the other biological sample (indicated on the top line of the columns), number of differentially methylated exons (DMEs; Fisher Exact Test *p* < 0.05 in both of biological replicates), number of differentially AS-skipped exons (DSEs), number of overlap exons between DMEs and DSEs, and representation factors, which is the ratio between the number of observed overlapped exons with that of the expected one. A representation factor < 1 means that it is under-representated. *Denote *p* value strength. **c** Scatter plots of delta PSI (ΔPSI) of exons between embryo and indicated samples versus percent-methylation-change of exons in the same pairwise samples. Blue dots include values of all methylated exons, and red dots include values of only differentially skipped and methylated exons (i.e., DSEs and DMEs)

To investigate the association between intragenic methylation and RNA splicing in honey bee, we examined the distributions of various alternative splicing (AS) events—exon skipping, intron retention, and alternative 5′/3′ splicing—within methylated gene sequences across developmental stages. These distribution patterns show that no AS types correlate with the most highly methylated genic regions (Fig. 5a). Whereas methylation is particularly enriched within the second kb of gene sequences, all the examined AS profiles are relatively depleted from this region and enhanced either upstream or downstream (Fig. 5a). One interpretation of this result is that methylation suppresses AS, consistent with previous findings showing that genic methylation enhances the inclusion of alternatively spliced regions [44].

Next, we investigated whether developmentally associated gene body methylation dynamics are connected to differential levels of alternative splicing. For this purpose, we measured the number of differentially alternatively spliced skipped exons (DSEs) among all methylated exons, and compared this with the number of differentially methylated exons (DMEs) in paired developmental stages (Fig. 5b). DSEs overlapped DMEs significantly less than expected by random chance, with typically half of the expected number of DSEs corresponding to DMEs (Fig. 5b). This result indicates that the methylation level of most DSEs is stable during honey bee development. We next compared the levels of methylation of alternatively spliced exons and their level of inclusion in mature mRNA. To this end, we correlated percent-methylation-change of exons between two developmental stages against the delta of percent-spliced-in (PSI) of these exons. This analysis did not reveal a correlation of appreciable magnitude between methylation and developmentally regulated PSI changes (Fig. 5c). Overall, our results do not indicate that global fluctuations in gene body methylation promote alternative splicing during honey bee development.

### DNA methylation is distinctly regulated in the soma and germline

**Fig. 6:**
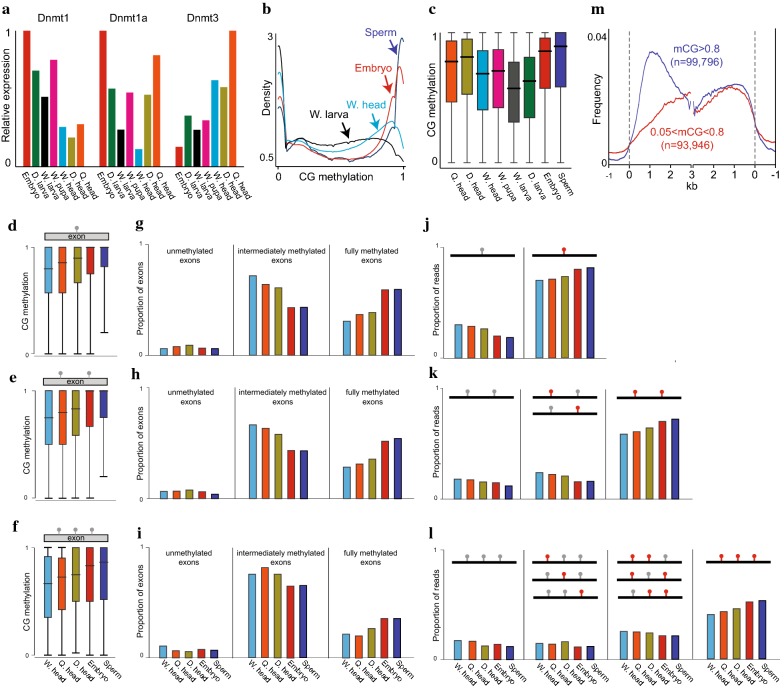
DNA methylation is distinctly regulated in the soma and germline. **a** Relative expression of DNMTs. FPKM values of DNMTs were normalized to the highest DNMT within each group. **b** Kernel density plot of methylation level of single cytosines in the indicated samples, which are methylated to at least 10% in either of the samples. **c** Boxplots of methylation level of single cytosines in the indicated samples derived from methylated CG sites (mCG > 0.5 in either of the samples). **d**–**f** Boxplots of methylation level of single, double, and triple CG containing exons that are methylated to at least 34% and with minimum coverage of 4 reads in at least one of the indicated samples. Lollipops represent CG sites. **g**–**i** Proportions of unmethylated (0 ≥ and < 0.1), intermediately methylated (0.1 > and < 0.9), and fully methylated (0.9 > and ≤ 1) exons (same exons as in **d**–**f**, respectively). **j**–**l** Proportions of single BS-seq reads aligned to the same exons as in **d**–**f**, respectively. Black lines represent single reads, red/gray lollipops represent methylated/unmethylated CGs, respectively. **m** Frequency of highly (mCG ≥ 0.8) and lowly (0.05 < mCG < 0.8) methylated CG sites were averaged in 20-bp bins along methylated genes (averaged genic methylation > 0.05) essentially as described in Fig. 1a

To explain the molecular mechanism of gene body methylation dynamics during honey bee development, we examined the expression of honey bee DNA methyltransferases. The honey bee genome encodes two Dnmt1 CG methylation maintenance enzymes and one Dnmt3 de novo methyltransferase [60]. Our RNA-seq data showed that all honey bee Dnmts are expressed and differentially regulated during development (Fig. 6a). Both Dnmt1s are most highly expressed in the embryo (Fig. 6a). In contrast, Dnmt3 RNA level is the lowest in the embryo and consistently increases during development, being most enriched in adult heads (Fig. 6a). The highest expression level of both Dnmt1s in the embryo corresponds with the highest CG methylation level during this stage, whereas the increase in CG methylation in post-larva stages corresponds with an increase in Dnmt3 expression (Figs. 1a, 6a). Accordingly, the drop in CG methylation in the larva could be explained by passive DNA demethylation due to a decrease in Dnmt1 expression, whereas the recovery in DNA methylation in the pupa and adult samples could be a result of increased Dnmt3 activity.

Consistent with the high expression of Dnmt1 enzymes in the embryo, this developmental stage shows more robust maintenance of CG methylation than any of the post-embryonic stages. Embryonic methylation at single CG cytosines shows a binary distribution: most cytosines are either not methylated or fully methylated (Fig. 6b). In comparison, cytosine methylation in postembryonic samples has a more gradual distribution with few completely methylated sites (Fig. 6b). The difference in the distribution and median level of CG methylation in the embryo versus post-embryonic samples is clear when only methylated cytosines are considered (Fig. 6c). Interestingly, these analyses also show an even stronger binary distribution of CG methylation in sperm cells, compared to the embryo (Fig. 6b), with the highest median methylation level (90%) among all samples (Fig. 6c). CG methylation in sperm is also the highest in complete exons containing single, double, and triple CG sites (which represent 84% of methylated exons) (Fig. 6d–f). Sperm and embryo have more fully methylated exons (> 90%) than partially methylated ones (> 0.1 and ≤ 0.9), whereas adult somatic samples have the opposite ratio (Fig. 6g–i). Finally, by analyzing methylation level in single BS-seq molecules, we found sperm to have the highest frequency of fully methylated reads and lowest frequency of partially and unmethylated reads (Fig. 6j–l). Overall, these results suggest that methylation in sperm is more efficient than in somatic tissues, both globally among all methylated exons in the genome as well as locally within intra-exonic neighboring CG sites.

Further examination of the distribution of CG sites along genes, differentiated by their methylation level in sperm, revealed that highly methylated CGs (mCG ≥ 0.8) are enriched in upstream genic sequences, whereas lowly methylated CGs (0.05 < mCG < 0.8) are depleted from these regions (Fig. 6m). The distribution pattern of highly methylated CGs in sperm matches that of overall CG methylation in bee tissues (Fig. 1b). Thus, the efficiency of sperm methylation and the probability of inheriting a methylated site may shape bee methylation patterns over generations.

### Non-CG methylation is a conserved regulator of animal nervous systems

**Fig. 7:**
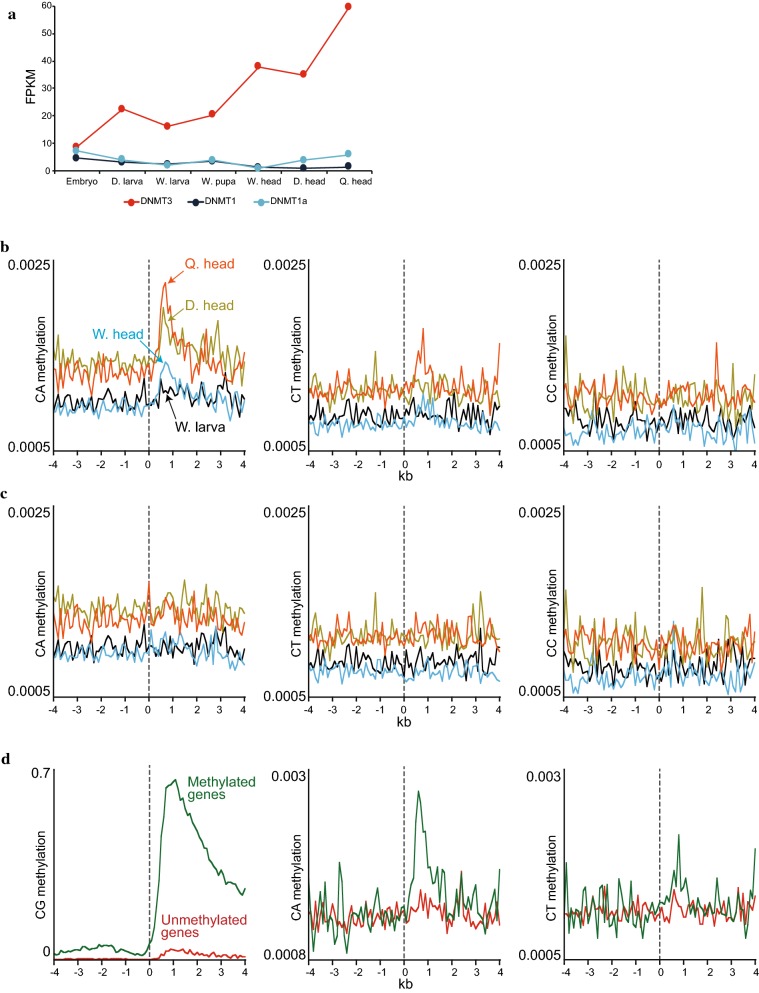
CW (CA or CT) methylation is enriched in adult honey bee heads. **a** Averaged FPKM values of DNMTs in the different biological samples. **b** Heads and W. larva CH methylation located specifically in exons was averaged in 100 bp bins along honey bee genes essentially as described in Fig. 1a. CH methylation of all developmental stages can be found in Additional file 1: Figure S5B. **c** Heads and W. larva CH methylation located specifically in introns was averaged in 100 bp bins along honey bee genes essentially as described in Fig. 1a. **d** Q. heads CG, CA, and CT methylation located specifically in exons were averaged in 100 bp bins along honey bee methylated (averaged genic CG methylation > 0.05) and unmethylated (averaged genic CG methylation < 0.005) genes essentially as described in Fig. 1a

Whereas the absolute expression levels of Dnmt1 and Dnmt1a are comparable, the transcript level of Dnmt3 is on average 18 and 11 times higher than that of Dnmt1 and Dnmt1a, respectively (Fig. 7a). This differs from mammals, in which Dnmt1 expression is usually higher than that of Dnmt3 [61]. Although most methylation in animals is concentrated in CG sites, recent reports showed that non-CG (CH, H = A, C or T) methylation also exists and is mostly targeted by Dnmt3 [7]. In mammals, CH Dnmt3-dependent methylation is particularly enriched in brain cells [7]. These findings prompted us to check whether the increase of Dnmt3 expression in honey bee heads is similarly associated with CH methylation. By examining methylation in genic sequences, we discovered an increase in CH methylation in adult heads (Fig. 7b and Additional file 1: Figure S5A). CH methylation was mostly enriched in queen heads, as compared to drone and worker heads (Fig. 7b and Additional file 1: Figure S5A), in accordance with the highest Dnmt3 expression level found in this sample (Figs. 6a, 7a). The increased CH signal is not due to nucleotide polymorphism between the sequenced samples and the reference genome, i.e., CG sites in reads that align to CH sites in the genome, as the incidents of such events were lower than 5% in any of the samples and the lowest in queen heads (1.68%), which have the highest CH methylation signal (Additional file 1: Figure S5B). CH methylation was particularly enriched on cytosines adjacent to adenine (CA), and to a lesser extent to thymine (CT), similar to mammalian CH methylation context preferences [7]. CA methylated sites were not found to be further enriched within a particular motif, such as TACAC in glia and neuron cells (Additional file 1: Figure S5C) [7]. Finally, CW (W = A or T) methylation in the gene bodies of adult heads is specifically enriched in genes that also have CG methylation (Fig. 7d) and is localized in the same genic region (upstream exons) where CG methylation is highest (Fig. 7b, d), also similar to CH methylation in mammalian brain tissue [7]. Overall, while CH methylation in bee heads is low (~ 0.2%), its signal is continuous (~ 200 bp) and above the noise level, associated with Dnmt3 expression, and enriched in a particular context (CA), tissue (heads), and genes (CG methylated), indicating that it is a real biological signal and implying that increased CH methylation during brain development is an ancient epigenetic phenomenon shared by insects and mammals.

## Discussion

**Fig. 8:**
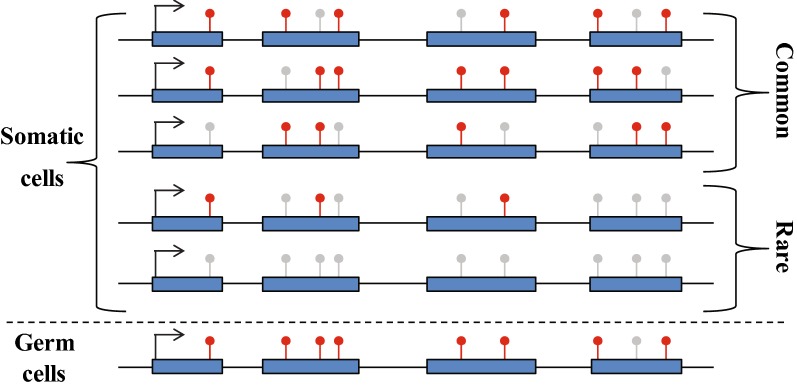
Schematic illustration of gene body methylation dynamics in honey bee somatic and germ cells. In germ cells, gene body CG methylation is highly maintained especially in upstream genic regions. In somatic cells, CG methylation is commonly fluctuated at individual CG sites but very rarely at the level of full exons or complete genes. Blue boxes represent exons; black lines represent intronic or intergenic sequences; and red and gray ‘lollipops’ represent methylated and unmethylated CG sites, respectively

We show that gene body methylation globally fluctuates during honey bee development, yet the patterns of methylation remain essentially unchanged from sperm to adult (Figs. 1, 2, and 8). Developmentally robust patterns of gene body methylation have been proposed in ants [58] and in the invertebrate chordate *Ciona intestinalis* [16], and are also the norm in plants [62–64], suggesting that developmental stability of gene body methylation may be ancient and conserved.

We find the highest levels of methylation in bee sperm, whereas methylation fidelity in somatic tissues is considerably lower (Fig. 6). More efficient maintenance of methylation in male and female gametes has also been reported in flowering plants [65, 66]. Plants have robust transgenerational inheritance of DNA methylation [4, 67, 68], which necessitates efficient preservation of methylation patterns in reproductive tissues. The high efficiency of DNA methylation in sperm suggests that DNA methylation is also transgenerationally inherited in bees. Consistent with this hypothesis, chromosomes in hybrid wasps faithfully maintain their ancestral methylation patterns [59]. Thus, transgenerational epigenetic inheritance of DNA methylation may be a common feature of invertebrates and plants.

There could be a number of molecular mechanisms regulating methylation fidelity during development. Our data suggest that alterations in the expression of DNMT1 and DNMT3 (Fig. 6a) could play a role in enhancing, or passively reducing, the efficiency of maintenance (DNMT1) and de novo (DNMT3) DNA methylation activities during honey bee development. Alternatively or additionally, methylation fidelity could be regulated biochemically via DNMT cofactors, e.g. UHRF1 [69], or through the involvement of active DNA demethylation pathways [70], which are yet to be explored in honey bee.

Except for adult heads, we did not find any significant associations between methylation dynamics and any transcriptional or posttranscriptional alterations among various developmental stages (Figs. 4 and 5, and Table 1). Developmentally regulated genes also have a strong tendency not to be methylated, as has been observed in many animal and plant species [14–28]. We, therefore, propose that for the most part, gene body methylation is not involved in regulating transcriptional changes during honey bee development. Instead, gene body methylation is likely to have a homeostatic function in maintaining normal gene expression, for example by suppressing intergenic transcript initiation [51]. Alternatively, genic methylation might be involved in genomic activities unrelated to gene expression, such as genome structure or integrity [32]. A possible explanation for the global gene body methylation fluctuations during development is that the highest levels of methylation are not required for proper function. This hypothesis is based on the finding that evolutionary selection acts on entire regions of gene body methylation, rather than individual cytosines [71]. In somatic cells with limited division potential, imperfect maintenance of methylation may, therefore, preserve functionality if methylation of an entire exon or gene is maintained above a threshold level.

Our finding that differential methylation and gene expression are correlated in comparisons involving adult heads (Table 1) may relate to non-CG methylation. Bee CH methylation is specifically targeted to CW sites, associated with Dnmt3 expression, localized in CG methylated regions, and particularly enriched in adult heads (Fig. 7). These characteristics are similar to CH methylation specificities described in mammals, and CH methylation was shown to regulate transcription and differentiation of human neurons [7]. Similar regulation may occur in honey bee, and would be consistent with the finding that knockdown of Dnmt3 caused worker larvae to develop into queens [55]. Therefore, tissue-specific fluctuations in the overall levels of gene body methylation, coupled with variable non-CG methylation, may regulate the expression of certain genes and be important for normal development. However, our findings strongly argue that this type of regulation is unlikely to be the core function of gene body methylation.

## Conclusions

We demonstrate that honey bee DNA methylation is distinctly regulated in the soma and germline. In the germline, methylation is efficiently maintained, likely to preserve methylation patterns across generations. In contrast, methylation can fluctuate in somatic cells, as long as overall methylation of exons and genes remains at an adequate level. Genome-wide methylation dynamics do not associate with gene regulation, and most developmentally regulated gene expression alterations occur in unmethylated genes. Accordingly, we conclude that gene body methylation is required for the homeostasis of gene expression and that global fluctuation of genic methylation is a non-functional feature of cells with limited division potential. Finally, our data suggest that heightened non-CG methylation is a conserved regulator of animal nervous systems, which may influence gene expression and development.

## Methods

### Biological samples

European honey bees were grown in Langstroth hives at the Harry H. Laidlaw Jr. Honey Bee Research Facility at University of California Davis. Honey bee samples were collected during summer in two rounds separated about 3 weeks apart (for two biological replicates). Worker embryos (eggs) were transferred from honeycomb cells into microcentrifuge tubes (50 per/tube). Drone and worker larvae were obtained at about 3rd and 4th instar developmental stages. Worker pupae were collected at about pink eyes developmental stage. Worker heads were collected from bees newly emerged from their own growing cells. Drone heads were collected from the same colony as drone larvae and worker embryos, larvae, pupae, and adult heads were collected from. Semen was collected from mature drones essentially as described in [72]. All samples were flash frozen in liquid nitrogen immediately upon collection. Except for queen heads, for which we used a single head per experiment, the rest of the biological sampling consisted of a mix of individuals, three larvae/pupae/heads, 50 embryos, and 20ul of semen.

### Bisulfite sequencing (BS-Seq)

About 500 ng of genomic DNA was isolated from various honey bee developmental stages, fragmented by sonication, end repaired, and ligated to custom synthesized methylated adapters (Eurofins MWG Operon) according to the manufacturer’s (Illumina) instructions for gDNA library construction. Adaptor-ligated libraries were subjected to two successive treatments of sodium bisulfite conversion using the EpiTect Bisulfite kit (QIAGEN), as outlined in the manufacturer’s instructions. The bisulfite-converted libraries were then amplified by PCR using ExTaq DNA polymerase (Takara Bio) for 12–14 cycles. The enriched libraries were purified using the solid-phase reversible immobilization method using AM-Pure beads (Beckman Coulter) prior to quantification with a Bioanalyzer (Agilent). Sequencing on the Illumina GAII and HiSeq 2000 platform was performed at the Vincent J. Coates Genomic Sequencing Laboratory at the University of California, Berkeley, USA (UC Berkeley) to generate single-end 76 and 100 base reads.

### RNA-Seq

Total RNA samples were isolated using the RNeasy mini kit (QIAGEN #74106) including on-column DNase treatment. mRNA was purified from 10 to 50 μg of total RNA by two cycles of poly-A enrichment using the Oligotex kit (QIAGEN #72022) followed by an rRNA removal step using the RiboMinus Eukaryote Kit for RNA-Seq (Invitrogen #A1083702). Precipitated mRNA samples were eluted with 9 ml of RNase-free water and fragmented with 1 ml of fragmentation buffer (Ambion, #AM8740) at 70 °C. Reactions were stopped after 5 min by adding 1-ml stop buffer, and RNA was purified by ethanol precipitation. cDNA was synthesized from 100 to 300 ng of mRNA using SuperScript III reverse transcriptase (Invitrogen #18080-051). Double-stranded DNA was synthesized according to the instructions using the SuperScript Double-Stranded cDNA Synthesis Kit (Invitrogen). DNA was cleaned with a QIAquick PCR spin column (QIAGEN, #28106), sequencing adapters were ligated according to the Illumina protocol, and the library was amplified by 18 cycles of PCR using Phusion DNA polymerase (NEB, #F-530). Bands around 300 bp were gel purified and libraries were sequenced on the Illumina HiSeq 2000 platform at the Vincent J. Coates Genomic Sequencing Laboratory at UC Berkeley to generate single-end 100 base reads.

### Data analysis

#### Differential methylation

Identifying differential methylation between various samples was performed by Fisher Exact Test using the number of Cs (methylated cytosines) and Ts (unmethylated cytosines) of any pair of samples. A site was determined to be differentially methylated if its *p* value was lower than 0.001 in both biological replicates.

#### Percent-methylation-change

This number was calculated by dividing the difference in methylation level between two samples by the level of methylation in the sample with the higher methylation level. For example, percent-methylation-change between embryo and W. larva was calculated as follows:

$$ \frac{{{\text{Embryo}} \;{\text{mCG}} - {\text{W}}.{\text{larva}}\;{\text{mCG}}}}{{{\text{Embryo}} \;{\text{mCG}}}} \;\;{\text{if}}\;{\text{Embryo}}\;{\text{mCG}} > {\text{W}}.{\text{larva}}\; {\text{mCG}} $$

$$ - \frac{{{\text{W}}.{\text{larva}}\;\;{\text{mCG}} - {\text{Embryo}}\; {\text{mCG}}}}{{{\text{W}}.{\text{larva}}\; {\text{mCG}}}}\;{\text{if}}\;{\text{Embryo}}\;{\text{mCG}} < {\text{W}}.{\text{larva}}\; {\text{mCG}} $$

#### Kernel density plots

Kernel density plots compare percent-methylation-change within either single-Cs, exons, or genes. For comparisons, we used genomic sites with at least 10 and 20 informative sequenced cytosines for single-C and complete exons or genes, respectively. Additionally, we used genomic sites with fractional methylation of at least 0.1 in at least one of the samples being compared.

#### Hierarchical clustering

The hierarchical clustering was performed and visualized in heatmaps using clustermap function from the Python seaborn library, with Euclidean distance matrix and average linkage method.

#### Gene expression

Raw Illumina RNA-Seq 100 base reads were first mapped to the most recent honey bee genome assembly (Amel_4.5; [73]) using Tophat [74], with the following changes to the default Tophat v2.0.1 parameters –I 100,000 (maximum intron length) and –no-novel-juncs (limiting the alignment to v3.2 honey gene annotation). Gene expression abundance and changes were calculated using the Cufflinks and Cuffdiff softwares [74] based on the honeybee 4.5 genome, as well as the v3.2 gene annotation file and default parameters with the addition of –min-reps-for-js-test. Differential expression was considered as statistically significant when the *q*-value (FDR correction) was lower than 0.05 and the FPKM fold change between two samples was higher than two.

#### Gene responsiveness

We calculated gene responsiveness per annotated gene by counting the number of times it was differentially expressed within the seven developmental stages, i.e., 21 pairwise comparisons in total. Accordingly, a gene that was not differentially expressed in any of the pairwise comparisons, i.e., was similarly expressed in all developmental stages, was given a gene responsiveness score of zero. The maximum level of gene responsiveness in our data is 21, which was given to genes that were found to be differentially expressed in all 21 pairwise comparisons.

#### Significance of overlap between differentially methylated and alternatively expressed genes

*RNA splicing*: Alternative splicing events, and subsequently differential alternative splicing events, were identified by first running Cufflinks (version 2.2.1) on each sample and then Cuffmerge (version 2.2.1) to find novel transcript isoforms with –min-intron-length 70 –max-intron-length 100,000 settings [75]. To quantify transcript isoform level expression, the transcriptome annotation file from Cuffmerge was transformed into a transcriptome fasta file (using the gffread tool in Cufflinks version 2.2.1) and Kallisto (version 0.42.3) was run with fastq files as input (fragment length parameter − l set to 200) [76]. Transcripts with an expression of less than 2 transcripts per million (TPM) were then removed. The SUPPA program [77] was used to analyze alternative splicing. Using the Cuffmerge GTF, SUPPA identifies all alternative events in the transcriptome, and then uses transcript abundances calculated by Kallisto to find the percentage spliced in (PSI) of each alternative event in each sample. Average PSI values were then calculated for each event between the two biological replicates and a t-test with multiple correction testing was performed to find differential alternative splicing. Differential events were defined by having a delta PSI of >=0.1 and a *q*-value of < 0.05.

## Supplementary information

**Additional file 1: Figure S1.** Experimental reproducibility of gene body methylation dynamics during honey bee development. (A) Averaged CG methylation levels in the specified annotations, calculated separately for each of the biological replicates. Methylated exons (Me. exons) are exons with a minimum of 10% methylation in either of the samples. The y-axis is broken into two linear scales 0–10% and 20–50%. (B) Patterns of CG methylation in gene bodies within exonic sequences (i.e. excluding introns) separated to experiments. CG methylation profiles were generated similar to Fig. 1c. (C, D) Genomic snapshots of CG methylation of each of the replicates in a large-scale genomic region (D) and zoom in on single exons (E). **Figure S2.** Correlation of exon methylation between biological replicates. Density scatter plots of methylation level in methylated exons (defined in S1A) correlated between each of the biological replicates. Note the high signal at maximum methylation in sperm and embryo samples. r is Pearson correlation coefficient value (*p* < 10^−4^ for all correlations). **Figure S3.** Gene body methylation is robustly maintained during honey bee development. (A) Bar graphs of the total number of partially and fully differentially methylated cytosines (left panel), exons (middle panel), and genes (right panel) between embryo and indicated developmental stages. (B) Sunflower plots (density scatter plot) of percent-methylation-change of individual CG cytosines between two biological replicates. The top right corner enclosed by dashed lines, holds the fDMC sites (mCG ≥ 0.9). Note the single hexagon on the top right corner of the graphs containing multiple dark lines, which sum to 22–44% out of total cytosines in the graphs. (C) Kernel density plots of percent-methylation-change between averaged methylation in embryo versus indicated samples calculated for exons or genes separated to experiment 1 and experiment 2. Genes and exons were selected for the analysis if their average CG methylation was at least 10% in either of the biological stages in the relative experiment. (D) Scatter plots of percent-methylation-change in exons (upper plots) or genes (lower plots) between two comparison of embryo vs. other indicated samples. *r* is Pearson correlation coefficient value (*p* < 10^−4^ for all correlations). **Figure S4.** Gene body methylation dynamics are not associated with developmentally regulated transcriptional profiles. (A) Sunflower plot of LogFC of RNA reads between embryo and indicated samples versus averaged genic CG methylation in embryos. obs. equals observations. (B) Scatter plots of LogFC of RNA versus percent-methylation-change between indicated samples. Red dots are of only genes that were found to be both differentially methylated (Fisher exact test *p* < 0.05 in both experiments) and alternatively expressed (*T* test *p* < 0.05 and FC > 2). ‘*r*’ represents Pearson Correlation Coefficient values. **Figure S5.** CW methylation is enriched in adult honey bee heads. (A) CH methylation of all sequences (left) or specifically located in exons (middle) or introns (right) were averaged in 100 bp bins along honey bee genes essentially as described in Fig. 1a. (B) Quantification of CH error rate in each of the samples which is derived from methylated CG sites in reads overlapping CH sites in the reference genome. (C) Representative methylated CA motifs from queen bee heads. The relative frequencies of nucleotides around the methylated cytosine are represented through the Python seqlogo script. The height of each letter represents its information content.

## Data Availability

The raw and processed sequencing data generated in this study have been submitted to the NCBI Gene. Expression Omnibus (GEO; https://www.ncbi.nlm.nih.gov/geo) under accession numbers of GSE116629.
