# Supplementary material for: DNA methylation is maintained with high fidelity in the honey bee germline and exhibits global non-functional fluctuations during somatic development
Source: Epigenetics Chromatin. 2019 Oct 10;12:62. doi: 10.1186/s13072-019-0307-4 (PMC6786280; doi:10.1186/s13072-019-0307-4)
Supplement: Supplementary file 1 — Additional file 1: Figure S1. Experimental reproducibility of gene body methylation dynamics during honey bee development. (A) Averaged CG methylation levels in the specified annotations, calculated separately for each of the biological replicates. Methylated exons (Me. exons) are exons with a minimum of 10% methylation in either of the samples. The y-axis is broken into two linear scales 0–10% and 20–50%. (B) Patterns of CG methylation in gene bodies within exonic sequences (i.e. excluding introns) separated to experiments. CG methylation profiles were generated similar to Fig. 1c. (C, D) Genomic snapshots of CG methylation of each of the replicates in a large-scale genomic region (D) and zoom in on single exons (E). Figure S2. Correlation of exon methylation between biological replicates. Density scatter plots of methylation level in methylated exons (defined in S1A) correlated between each of the biological replicates. Note the high signal at maximum methylation in sperm and embryo samples. r is Pearson correlation coefficient value (p < 10−4 for all correlations). Figure S3. Gene body methylation is robustly maintained during honey bee development. (A) Bar graphs of the total number of partially and fully differentially methylated cytosines (left panel), exons (middle panel), and genes (right panel) between embryo and indicated developmental stages. (B) Sunflower plots (density scatter plot) of percent-methylation-change of individual CG cytosines between two biological replicates. The top right corner enclosed by dashed lines, holds the fDMC sites (mCG ≥ 0.9). Note the single hexagon on the top right corner of the graphs containing multiple dark lines, which sum to 22–44% out of total cytosines in the graphs. (C) Kernel density plots of percent-methylation-change between averaged methylation in embryo versus indicated samples calculated for exons or genes separated to experiment 1 and experiment 2. Genes and exons were selected for the analysis if their average [file 13072_2019_307_MOESM1_ESM.pdf]

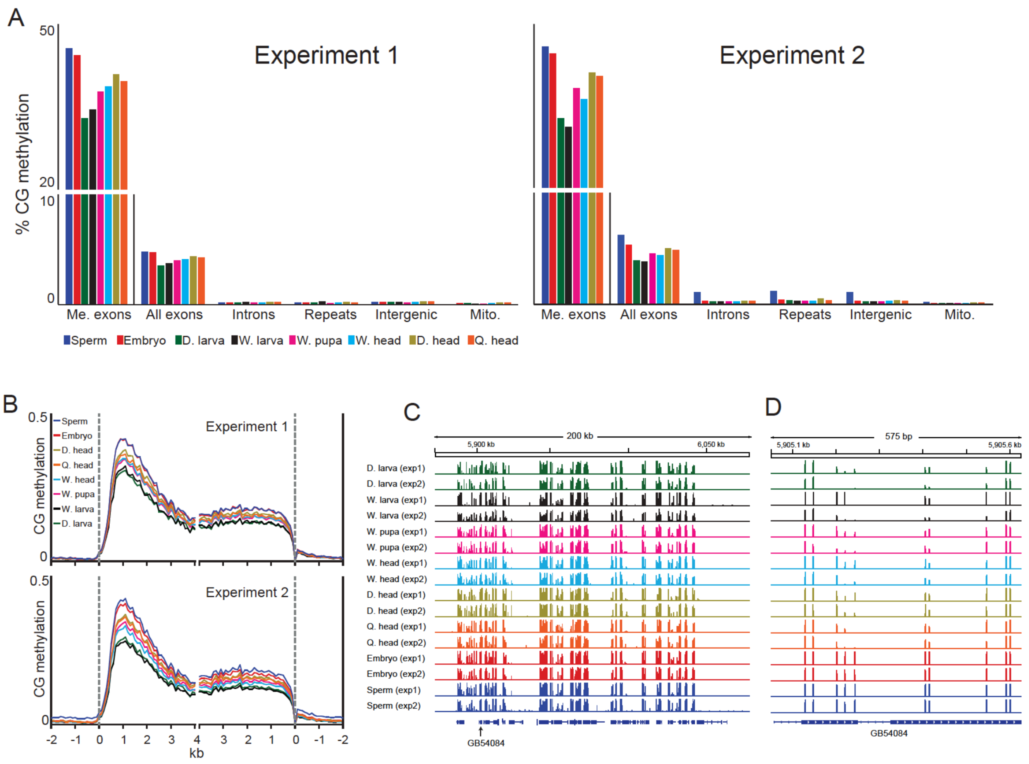

Figure S1. Experimental reproducibility of gene body methylation dynamics during honey bee development

(A) Averaged CG methylation levels in the specified annotations, calculated separately for each of the biological replicates. Methylated exons (Me. exons) are exons with a minimum of 10% methylation in either of the samples. The y-axis is broken into two linear scales 0-10% and 20-50%. (B) Patterns of CG methylation in gene bodies within exonic sequences (i.e. excluding introns) separated to experiments. CG methylation profiles were generated similar to Figure 1C. (C-D) Genomic snapshots of CG methylation of each of the replicates in a large scale genomic region (D) and zoom in on single exons (E).

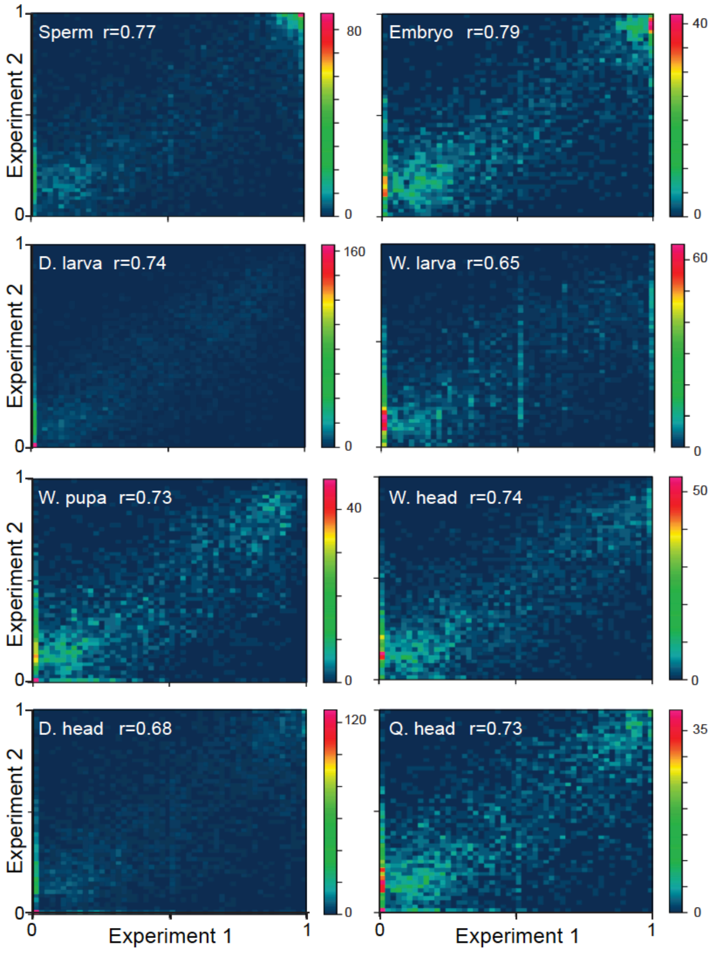

Figure S2. Correlation of exon methylation between biological replicates
Density scatter plots of methylation level in methylated exons (defined in S1A) correlated between each of the biological replicates. Note the high signal at maximum methylation in sperm and embryo samples. r is Pearson correlation coefficient value ($p<10^{-4}$ for all correlations).

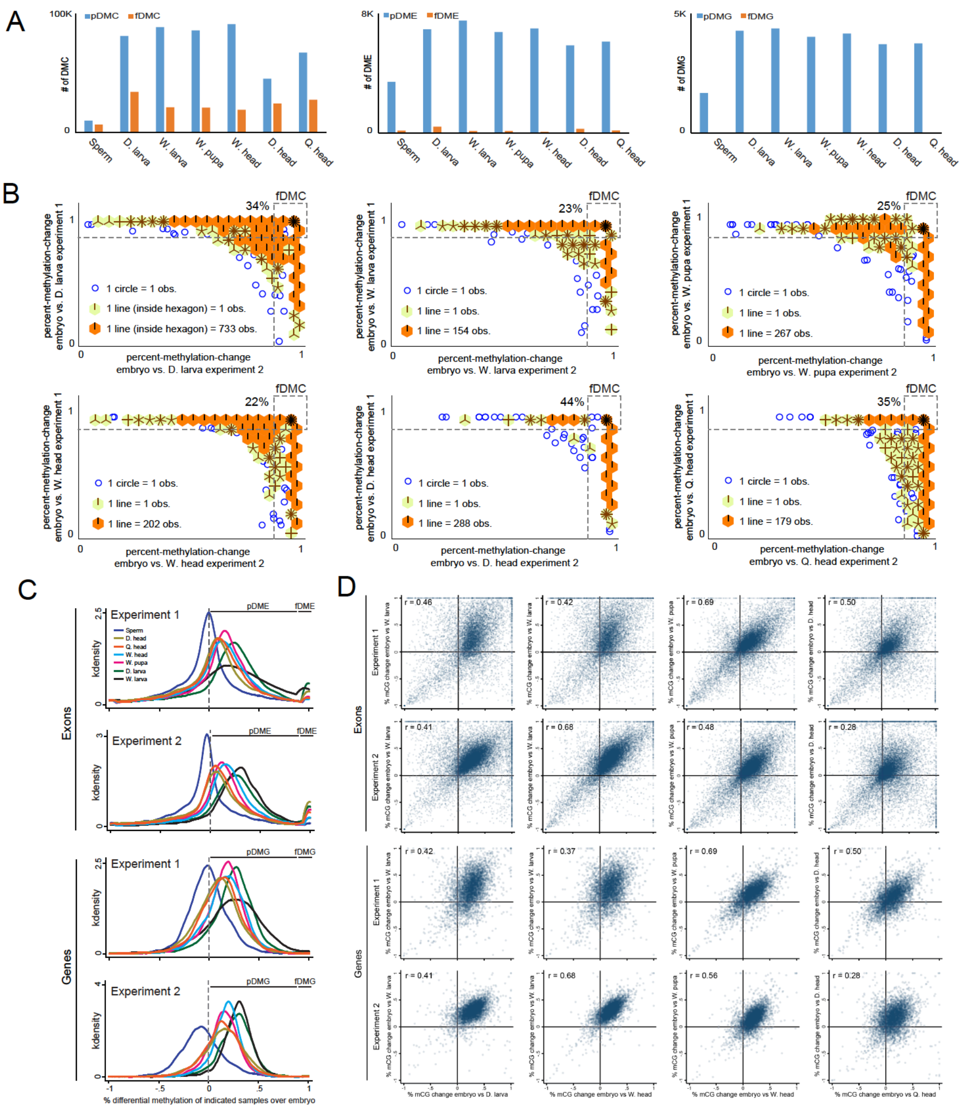

Figure S3. Gene body methylation is robustly maintained during honey bee development
(A) Bar graphs of the total number of partially and fully differentially methylated cytosines (left panel), exons (middle panel), and genes (right panel) between embryo and indicated developmental stages. (B) Sunflower plots (density scatter plot) of percent-methylation-change of individual CG cytosines between two biological replicates. The top right corner enclosed by dashed lines, holds the fDMC sites (mCG ≥ 0.9). Note the single hexagon on the top right corner of the graph containing multiple dark lines, which sum to 22-44% out of total cytosines in the graphs. (C) Kernel density plots of percent methylation change between averaged methylation in embryo versus indicated samples calculated for exons or genes separated to experiment 1 and experiment 2. Genes and exons were selected for the analysis if their average CG methylation was at least 10% in either of the biological stages in the relative experiment. (D) Scatter plots of percent-methylation-change in genes (upper plots) or exons (lower plots) between two comparison of embryo vs. other indicated samples. r is Pearson correlation coefficient value ($p<10^{-4}$ for all correlations).

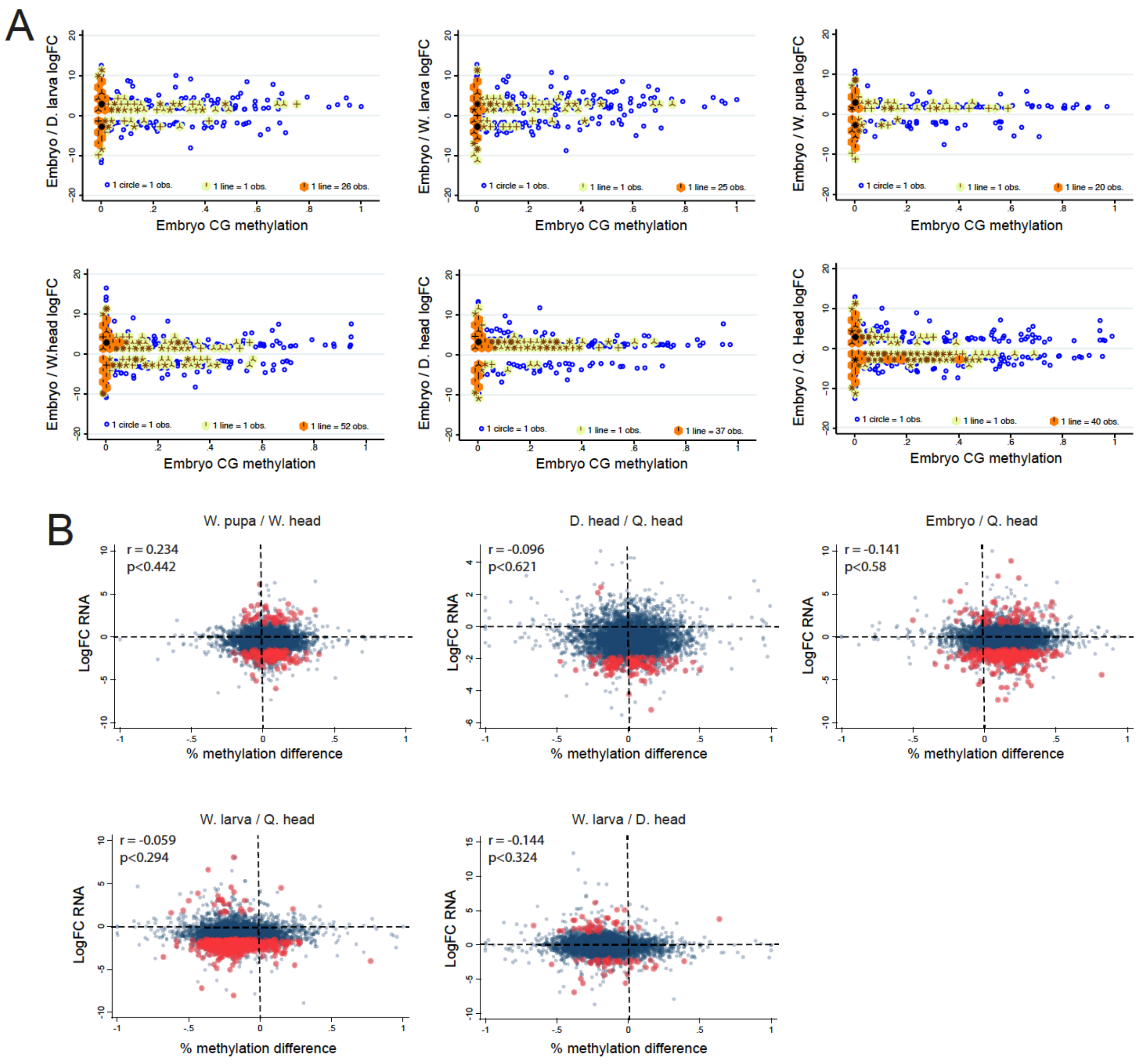

Figure S4. Gene body methylation dynamics are not associated with developmentally regulated transcriptional profiles

(A) Sunflower plot of LogFC of RNA reads between embryo and indicated samples versus averaged genic CG methylation in embryos . obs. equals observations. (B) Scatter plots of LogFC of RNA versus percent-methylation-change between indicated samples. Red dots are of only genes that were found to be both differentially methylated (Fisher exact test $p<0.05$ in both experiments) and alternatively expressed (test $p<0.05$ and $FC>2$). ‘r’ represents Pearson Correlation Coefficient values.

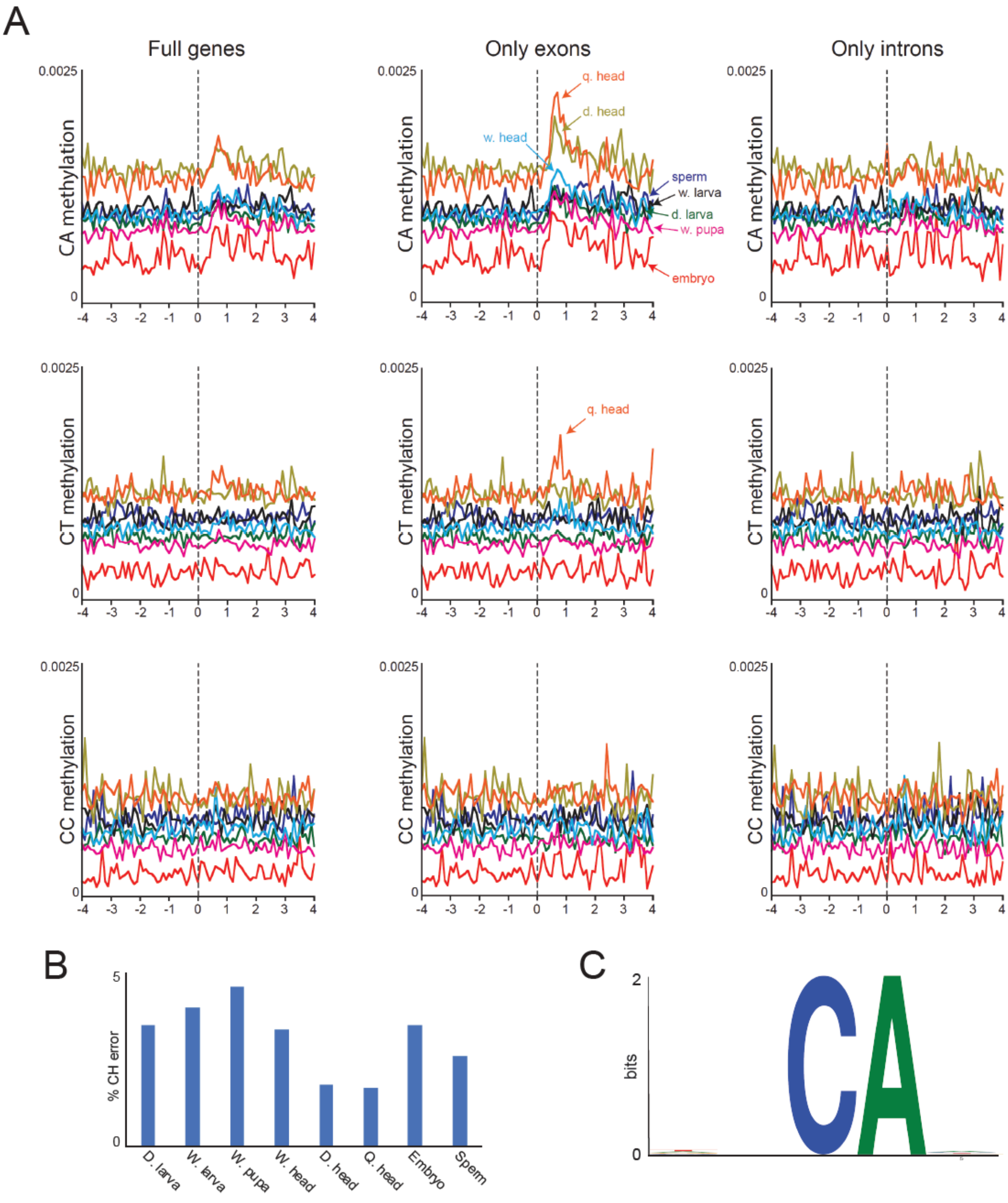

Figure S5. CW methylation is enriched in adult honey bee heads
(A) CH methylation of all sequences (left) or specifically located in exons (middle) or introns (right) were averaged in 100 bp bins along honey bee genes essentially as described in Figure 1A. (B) Quantification of CH error rate in each of the samples which is derived from methylated CG sites in reads overlapping CH sites in the reference genome. (C) Representative methylated CA motifs from queen bee heads. The relative frequencies of nucleotides around the methylated cytosine are represented through the seqlogo script. The height of each letter represents its information content.
